# Amino Acid Signaling in Skeletal Muscle Is Blunted by Prematurity in a Piglet Model

**DOI:** 10.1016/j.tjnut.2025.101303

**Published:** 2025-12-27

**Authors:** Antonio C Ramos dos Santos, Agus Suryawan, Ki Beom Jang, Rosemarie D Parada, Mahmoud A Mohammad, Marta L Fiorotto, Teresa A Davis

**Affiliations:** 1 Institute for Advancing Health Through Agriculture, Texas A&M AgriLife Research, College Station, TX, United States; 2 United States Department of Agriculture/Agricultural Research Service Children’s Nutrition Research Center, Department of Pediatrics, Baylor College of Medicine, Houston, TX, United States; 3 Department of Agricultural Biotechnology, Research Institute of Agriculture and Life Science, Seoul National University, Seoul, South Korea; 4 Department of Nutrition, Texas A&M University, College Station, TX, United States

**Keywords:** preterm, protein synthesis, mTOR, neonate, leucine

## Abstract

**Background:**

Preterm (PT) infants are at increased risk for reduced postnatal lean mass accretion. We established that the feeding-induced stimulation of protein synthesis in skeletal muscle is blunted in piglets born PT compared with those born at term.

**Objectives:**

We evaluated the extent to which key components of the amino acid–sensing pathways that regulate mechanistic target of rapamycin complex 1 (mTORC1) activation contribute to anabolic resistance in skeletal muscle of piglets born PT compared with those born term.

**Methods:**

Piglets delivered by cesarean section 10 d PT (*n* = 23) or at term (*n* = 22) were administered total parenteral nutrition for 3 d. On day 4, euinsulinemic-euaminoacidemic-euglycemic (FAST group), hyperinsulinemic-euaminoacidemic-euglycemic (INS group), or euinsulinemic-hyperaminoacidemic-euglycemic (AA group) clamps were performed for 2 h. Abundances and activation of amino acid signaling components in skeletal muscle were analyzed by immunoblotting.

**Results:**

Abundances of amino acid transporters LAT1/SLC7A5 (leucine), SLC38A9 (arginine), and SNAT2/SLC38A2 (glutamine) were unaffected by prematurity. Sestrin1- and Sestrin2-GATOR2 abundances were reduced (*P* < 0.05) by AA, consistent with leucine-induced dissociation of these inhibitory complexes; prematurity blunted this effect for Sestrin1-GATOR2 (*P* < 0.05). SAR1B, but not LARS-mTOR, leucine-sensor abundances were lower in PT than term animals (*P* < 0.05). TARS2 (threonine) and RAB1A (branched-chain amino acid) sensor abundances were lower in PT (*P* < 0.05). Arginine (CASTOR1-GATOR2), methionine (SAMTOR-GATOR1), and glutamine (ARF1) sensor abundances were unaffected by prematurity. AA-induced formations of RagA- and RagC-mTOR complexes were attenuated in PT compared with term piglets (*P* < 0.05). Both AA and INS stimulated mTORC1 phosphorylation, but these effects were blunted by prematurity.

**Conclusions:**

PT birth impairs the abundance and activation of multiple amino acid–sensing components upstream of mTORC1 in skeletal muscle. This disruption attenuates amino acid–induced mTORC1-dependent translation initiation and protein synthesis and likely contributes to the anabolic resistance, reduced lean mass, and extrauterine growth faltering frequently observed in premature infants.

## Introduction

Preterm (PT) birth is a global problem that affects the morbidity and survival of ∼9 million infants annually [1] and is the leading cause of death for children under 5 y of age [2]. Premature infants are at increased risk of adverse long-term health outcomes, including obesity, type 2 diabetes, and cardiovascular complications such as stroke and heart failure [3]. At term equivalent age, PT infants have a lower lean body mass and higher body fat than their term-born infant counterparts [4]. Because higher lean body mass is positively correlated with long-term health [5], better nutritional management is a crucial part of supportive care for PT infants.

Skeletal muscle, which constitutes the largest portion of lean body mass, is the fastest-growing protein mass in the body during the neonatal period [6]. This rapid skeletal muscle growth is attained primarily in response to its high sensitivity to the anabolic stimulation by postprandial concentrations of insulin and amino acids [7]. Using PT piglets, a relevant translational model for PT infants [8,9], we previously demonstrated that premature birth attenuates the postprandial activation of insulin- and amino acid–mediated signaling pathways that regulate mechanistic target of rapamycin complex (mTORC)1-dependent translation initiation and protein synthesis in skeletal muscle [10]. Recently, we showed that the activation of multiple insulin signaling components upstream of mTORC1 is reduced in PT piglets compared with those born at term [11]. These findings suggest that the blunted postprandial increase in muscle protein synthesis in PT piglets is due, at least in part, to impaired insulin-induced activation of mTORC1 and its downstream targets. However, the impact of prematurity on amino acid signaling pathways upstream of mTORC1 remains incompletely understood [10].

**FIGURE 1:**
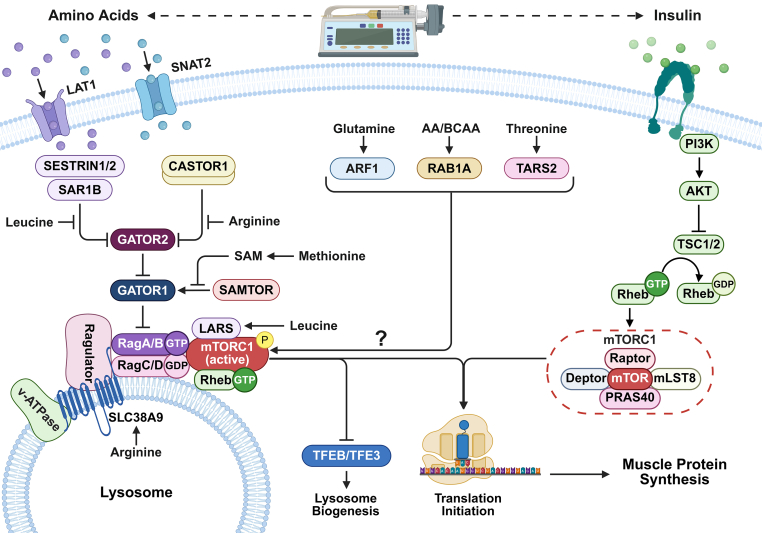
Current concepts of multiple amino acid–sensing input to mTORC1. AA, amino acid; Akt, RAC-α serine/threonine-protein kinase; ARF1, ADP-ribosylation factor 1; BCAA, branched-chain amino acid; CASTOR1, cytosolic arginine sensor for mTORC1 subunit 1; GATOR1/2, GAP activity toward RAGs 1/2; LARS, leucine-tRNA-synthase; LAT1/SLC7A5, L-type amino acid transporter 1; mTORC1, mechanistic target of rapamycin complex 1; PI3K, phosphatidylinositol-3-kinase; RAB1A, Ras-related protein Rab-1A; Rag A/B C/D, RAS-related GTP-binding protein A/B C/D; Ragulator, Late endosomal/lysosomal adaptor and MAPK and mTOR activator; Rheb, Ras homolog enriched in brain; SAM, S-adenosyl methionine; SAMTOR, S-adenosylmethionine sensor upstream of mTORC1; SAR1B, secretion-associated Ras-related GTPase 1B; Sestrin1/2, stress response protein 1/2; SLC38A9, solute carrier family 38 member 9; SNAT2, sodium-coupled neutral amino acid transporter 2; TARS2, threonyl-tRNA synthetase 2; TFEB, transcription factor EB; TSC1/2, tuberous sclerosis complex 1/2; V-ATPase, vacuolar H^+^-ATPase. Created in BioRender. Ramos dos Santos, A.C. (2026) https://BioRender.com/54b947z.

The molecular mechanisms by which insulin activates signaling cascades that control protein synthesis have been well-established [12]. In contrast, the mechanisms by which amino acids regulate mTORC1 activation in vivo are less understood [13,14]. Amino acids can act beyond their canonical function as primary substrates for protein synthesis by promoting the activation of novel signaling components crucial for recruiting mTORC1 to the lysosomal surface, where it is subsequently activated by Ras homolog enriched in brain (Rheb) [15]. Studies show that the mode of action of amino acids can depend on cell types and cellular context [[16], [17], [18]]. To date, only a limited number of amino acids have been identified as direct or indirect anabolic agents capable of enhancing mTORC1 activation and promoting protein synthesis [[19], [20], [21], [22], [23], [24], [25], [26], [27], [28], [29], [30]]. These include the branched-chain amino acids (BCAAs), methionine, arginine, threonine, and glutamine (Figure 1).

Leucine is recognized as the most potent anabolic amino acid, capable of directly modulating intracellular signaling pathways that regulate mTORC1 activation. Three cytosolic leucine sensors, stress response protein (Sestrin)1/2, secretion-associated Ras-related GTPase (SAR)1B, and leucine-tRNA-synthetase (LARS), have been identified as key mediators of leucine-induced mTORC1 signaling [19,20,26]. When leucine binds to Sestrin1/2 or SAR1B it disrupts their inhibitory interactions with the GAP activity toward RAG (GATOR)2, allowing GATOR2 to inhibit GATOR1 and promote mTORC1 recruitment and activation [19,20]. Alternatively, leucine can bind to LARS, enabling its interaction with RAS-related GTP-binding protein (Rag)D, which facilitates the recruitment and activation of mTORC1 [26,31]. In a cell culture study, it was shown that methionine can activate mTORC1, resulting in increased protein synthesis [32]. Mechanistically, methionine exerts its effects through its metabolite S-adenosyl methionine (SAM), which is sensed by SAMTOR (S-adenosylmethionine sensor upstream of mTORC1) [25]. SAMTOR functions as a negative regulator of mTORC1 by binding to the GATOR1-KICSTOR complex. Under methionine sufficiency conditions, SAM binds to SAMTOR and disrupts this interaction, thereby relieving inhibition and allowing mTORC1 activation [25].

In cell and animal models, arginine has been shown to act as a potent regulator of muscle protein metabolism by activating the mTORC1 signaling pathway and promoting nitric oxide production, ultimately enhancing protein accretion in skeletal muscle [24,33]. Mechanistically, arginine can activate mTORC1 by binding to its cytosolic arginine sensor, cytosolic arginine sensor for mTORC1 subunit 1 (CASTOR1), leading to the dissociation of CASTOR1 from the GATOR2 complex, which then inhibits GATOR1 and enables mTORC1 to translocate to the lysosome for activation [24]. Lysosomal amino acid transporter solute carrier family 38 member 9 (SLC38A9) is another putative arginine sensor; however, its mode of action is currently unknown [34]. Likewise, the exact molecular mechanisms by which ADP-ribosylation factor (ARF)1 (glutamine sensor), Ras-related protein Rab-1A (RAB1A; BCAA sensor), and threonyl-tRNA synthetase (TARS)2 (threonine sensor) have not been elucidated. Nevertheless, cellular biochemical or cellular function assays support the notion that these amino acid–sensing components contribute to mTORC1 activation [[27], [28], [29]].

The stimulation of skeletal muscle protein synthesis by postprandial increases in amino acids and insulin is attenuated in pigs born PT [10]. The extent to which amino acid–sensing pathways upstream of mTORC1 contribute to this impairment remains unclear. To address this question, we used an amino acid–clamp technique in PT piglets to isolate the specific effects of amino acids on mTORC1 signaling under controlled metabolic conditions [10]. In parallel, an insulin-clamp was performed to confirm that the stimulatory effect of insulin on mTORC1 activation occurs independently of amino acid signaling in PT muscle.

## Methods

### Animals, surgeries, and nutritional support

The experimental protocol was approved by the Institutional Animal Care and Use Committee, Baylor College of Medicine. All procedures were designed and conducted in accordance with the National Research Council’s Guide for the Care and Use of Laboratory Animals. The protocol was previously described in Rudar et al. [10]. Briefly, pregnant sows had ad libitum access to a commercial diet [Laboratory Porcine Grower Diet 5084; LabDiet; metabolizable energy (ME), 3160 kcal (ME)/kg diet as-fed; crude protein, 160 g/kg diet as-fed] and water. Surgeries were performed under general anesthesia using sterile techniques. Piglets (Yorkshire × Landrace × Duroc × Hampshire) at 103-d (PT; 3 litters) and 112-d (term; 2 litters) gestational age were delivered via cesarean section and placed in individual incubators maintained at a temperature between 29 and 32°C and on a 12-h light/12-h dark cycle. Piglets were injected with iron dextran (30 mg intramuscularly), received sterile sow plasma for passive immunity, and monitored and included in the study according to previously described criteria [10]. Piglets were surgically implanted with jugular vein and carotid artery catheters on the day of birth and monitored throughout the study. Parenteral nutrition, formulated to meet or exceed the nutrient requirements of neonatal pigs, was provided, as described in the studies by Rudar et al. [10,35]. Briefly, parenteral nutrition was administered initially at 6 mL/kg/h and increased to 8 and 10 mL/kg/h on days 2 and 3, respectively. All piglets on day 3 received the following: fluid, 240 mL/kg/d; glucose, 22 g/kg/d; amino acids, 16 g/kg/d; and lipid, 5 g/kg/d.

### Insulin and amino acid–clamp procedure

The clamp procedure has been described previously [10]. Briefly, after a 4.5-h fast, unanesthetized piglets were placed in a sling-restraint system and assigned randomly and unblinded (within each PT and term litter) to 1 of 3 treatment groups: *1*) euinsulinemic-euaminoacidemic-euglycemic clamp (FAST group); *2*) hyperinsulinemic-euaminoacidemic-euglycemic clamp (INS group); or *3*) euinsulinemic-hyperaminoacidemic-euglycemic clamp (AA group). This resulted in 6 experimental groups: PT-FAST (PT fasting, *n* = 7); PT-INS (PT insulin, *n* = 9); PT-AA (PT amino acid, *n* = 9); T-FAST (term fasting, *n* = 8); T-INS (term insulin, *n* = 9); and T-AA (term amino acid, *n* = 9). The basal concentrations of blood glucose and BCAA were determined 20 min before the start of the clamp procedure using the YSI 2300 STAT Plus (Yellow Springs Instruments) and a rapid enzymatic kinetic assay [36]. The clamp procedure was started with a primed, constant insulin infusion (Eli Lilly) at either 0 or 200 ng/kg^0.66^/min to maintain either fasting (euinsulinemic) or fed-state (hyperinsulinemic) plasma insulin concentrations. Blood was sampled from the jugular vein catheter every 5 min for 120 min and immediately analyzed for glucose and BCAA concentrations. Dextrose (Baxter Healthcare) was infused to maintain a glucose concentration within ±10% of each individual pig’s fasting value. To maintain the euaminoacidemia, an amino acid mixture [36] was infused at rates adjusted to keep the plasma BCAA concentration within 10% of each individual pig’s fasting level. Hyperaminoacidemia was achieved by infusing the same amino acid mixture at rates to mimic fed-state levels of BCAA concentrations.

### Plasma insulin and amino acids

Venous blood samples were collected immediately before initiating the clamp procedure (0 min), and at 30, 60, 90, and 120 min for later determination of plasma insulin and amino acid concentrations. Plasma was separated by centrifugation and stored at −20°C until analysis. Plasma insulin concentrations were determined with a commercial kit (Porcine Insulin Radioimmunoassay no. PI12K; MilliporeSigma). Plasma amino acid profiles were quantified using heated electrospray ionization liquid chromatography–mass spectrometry following derivatization to their dansylated derivatives and separation on a Kinetex 2.6 μm C18, 100 Å column (Phenomenex) [37]. A U-[^13^C^15^N] amino acid mixture, along with [^13^C_5_] citrulline and [^13^C_5_] ornithine (Cambridge Isotope Laboratories), served as internal standards. Calibration curves generated from serial dilutions of individual amino acids spiked with internal standards were used to calculate concentrations. The interassay and intraassay coefficients of variation were <4% for all amino acids, with the exception of ornithine (5.6% and 5.8%, respectively) and tyrosine (7.2% and 8.4%, respectively) [38].

### Immunoblots and immunoprecipitation assays

The assays were conducted using our previously described protocols [10]. In brief, frozen longissimus dorsi muscles were homogenized in ice-cold CHAPS (3-[[3-cholamidopropyl] dimethylammonio]-1-propanesulfonate) buffer containing protease and phosphatase inhibitors (Sigma Aldrich) and stored at −80°C. For immunoblotting, equal amounts of protein samples were subjected to electrophoresis, followed by electrotransfer onto PVDF membranes. The membranes were incubated overnight at 4°C with the appropriate primary antibodies, followed by a 1-h incubation with goat anti-mouse or anti-rabbit secondary antibodies (Cat. No. 170–6516 and 170–6515, respectively; Bio-Rad). Blots were developed using an enhanced chemiluminescence kit (Amersham) and visualized, and analyzed using a ChemiDoc-It Imaging System (UVP). The protein phosphorylation was normalized by dividing its values by the total abundance of the corresponding protein.

To determine protein–protein interactions, immunoprecipitations were performed using target-specific antibodies, followed by immunoblotting to detect specific associated target proteins [10]. Briefly, the supernatant containing 500 μg of protein was combined with appropriate antibodies and mixed on a platform rocker overnight at 4°C. After incubation, the immune complexes were captured by either a goat anti-mouse or anti-rabbit BioMag IgG (Cat. No. 84340-500 and 84300-500, respectively; Polysciences) bead slurry. The magnetic bead complexes were collected using a magnetic stand, washed twice with CHAPS buffer, and once with high-salt CHAPS buffer. The precipitates were rinsed with 100 μL of 1× SDS sample buffer, then boiled for 5 min, and centrifuged to collect the supernatant. The samples were immunoblotted with appropriate primary antibodies. The protein–protein complexes were normalized by the appropriate protein abundance of each protein target. Immunoblotting and immunoprecipitation assays were performed using the following primary antibodies: ARF1 (Cat. No. 68069-1-AP; ProteinTech Group), CASTOR1/GATSL3 (Cat. No. A13309; Boster Biological Technology), LARS (Cat. No. A304-316A; Bethyl Laboratories), Mios, a GATOR2 subunit (Cat. No. 13557; Cell Signaling Technology), mTOR phosphorylated Ser2448 (Cat. No. 2971; Cell Signaling Technology), mTOR total (Cat. No. 2972; Cell Signaling Technology), NPRL2, a GATOR1 subunit (Cat. No. 37344, 37344; Cell Signaling Technology), Rab1A (Cat. No. 13075; Cell Signaling Technology), RagA (Cat. No. 4357; Cell Signaling Technology), RagC (Cat. No. 9480; Cell Signaling Technology), Raptor (Cat. No. 2280; Cell Signaling Technology), SAMTOR/BMT2 (Cat. No. 21744-1-AP; ProteinTech Group), SAR1B (Cat. No. 22292-1-AP; ProteinTech Group), Sestrin1 (Cat. No. 55010-1-AP; ProteinTech Group), Sestrin2 (Cat. No. 66297-1-Ig; ProteinTech Group), SLC38A2/sodium-coupled neutral amino acid transporter (SNAT)2 (Cat. No. ARP33059_P050; Aviva Systems Biology), SLC38A9 (Cat. No. AAS59532C; Antibody Verify), LAT/1SLC7A5 (Cat. No. BMP011; MBL International), and TARS2 (Cat. No. 15067-1-AP; ProteinTech Group). Vinculin (Cat. No. A302-534A; Bethyl Laboratories) was used to adjust for variation in total loading.

### Statistical analysis

Power analysis indicated that 8 pigs/treatment group were needed to detect an absolute difference of 5%/d in skeletal muscle fractional protein synthesis rate, with an SD of 3.5%/d, type I error of 0.05, and power of 0.80. The fractional protein synthesis rate was selected for power calculation because it was the primary endpoint in the parent study [10] and directly reflected the anabolic response of skeletal muscle, ensuring adequate power to evaluate nutrient-induced activation of mTORC1 signaling. All statistical analyses were conducted using SAS software version 9.4 (SAS Institute). A 2-way analysis of variance was used to assess the fixed effects of gestational age at birth (GAB; PT or term), clamp condition (STATE; FAST, INS, or AA), and their interactions (GAB × STATE) on outcomes related to signaling proteins, plasma metabolites, and insulin concentrations. Individual pig was considered the experimental unit; pig and litter were included as random effects. All groups were represented in each litter. When significant main effects or interactions were detected, Tukey post hoc test was used to perform pairwise comparisons among treatment groups. Data are expressed as least-squares means ± SE. Statistical significance was defined as *P* ≤ 0.05.

## Results

### Hormones and metabolites

Circulating concentrations of insulin, BCAA, and glucose have been reported previously [10] and are briefly summarized in this study for context. To mimic the postprandial conditions [10], fasted piglets (4.5 h fast) were subjected to clamp procedures designed to elevate plasma insulin (∼100 μU/mL) or BCAA (∼1000 μmol/L), while maintaining euglycemia [10]. Target concentrations of insulin and BCAA were successfully achieved and sustained throughout the 60- to 120-min clamp period (Supplementary Table 1).

Plasma amino acid profiling revealed that concentrations of all indispensable and dispensable amino acids were elevated in the AA group than those in the FAST and INS groups (Table 1). Arginine was the only exception, showing a trend toward elevation in the AA group (*P* = 0.058), but not reaching statistical significance.

**TABLE 1:** Plasma concentrations of amino acids (expressed μmol/L) in preterm and term pigs during euinsulinemic-euaminoacidemic-euglycemic (FAST), hyperinsulinemic-euaminoacidemic-euglycemic (INS), or euinsulinemic-hyperaminoacidemic-euglycemic (AA) clamp conditions

Amino acids (μmol/L)	Preterm			Term			*P*		
	FAST	INS	AA	FAST	INS	AA	GAB	STATE	GAB × STATE
Indispensable	1708.6 ± 362.2^b^	1451.6 ± 319.4^b^	3814.7 ± 319.4^a^	1738.7 ± 338.8^b^	1673.1 ± 319.4^b^	4314.9 ± 319.4^a^	0.358	< 0.001	0.778
Arginine	192.3 ± 59.7	178.2 ± 52.6	370.1 ± 52.6	51.1 ± 55.8	69.2 ± 52.6	103.7 ± 52.6	< 0.001	0.058	0.302
Histidine	78.5 ± 21.9^bc^	65.7 ± 19.3^bc^	178.8 ± 19.3^a^	20.9 ± 20.5^c^	31.3 ± 19.3^c^	97.4 ± 19.3^b^	0.001	< 0.001	0.484
Isoleucine	93.5 ± 21.9^b^	87.5 ± 19.3^b^	317.9 ± 19.3^a^	61.8 ± 20.5^b^	76.9 ± 19.3^b^	342.6 ± 19.3^a^	0.719	< 0.001	0.374
Leucine	93.3 ± 22.7^b^	58.7 ± 20.1^b^	215.8 ± 20.1^a^	53.9 ± 21.3^b^	48.7 ± 20.1^b^	239.1 ± 20.1^a^	0.610	< 0.001	0.337
Lysine	407.9 ± 106.7^b^	354.9 ± 96.4^b^	932.1 ± 97.3^a^	244.1 ± 105.3^b^	286.1 ± 100.4^b^	858.7 ± 101.1^a^	0.309	< 0.001	0.846
Methionine	54.6 ± 15.6^c^	35.7 ± 13.7^c^	122.1 ± 13.7^b^	61.6 ± 14.6^c^	58.7 ± 13.7^c^	197.8 ± 13.7^a^	0.004	< 0.001	0.049
Phenylalanine	97.8 ± 15.8^b^	71.2 ± 13.9^b^	188.9 ± 13.9^a^	92.8 ± 14.8^b^	76.5 ± 13.9^b^	202.1 ± 13.9^a^	0.704	< 0.001	0.826
Threonine	407.9 ± 156.8^c^	350.5 ± 143.8^c^	769.4 ± 145.6^b^	943.3 ± 159.9^b^	784.9 ± 153.8^b^	1518.8 ± 154.9^a^	0.001	< 0.001	0.412
Tryptophan	37.5 ± 5.6^b^	30.3 ± 5.0^b^	70.7 ± 5.0^a^	27.5 ± 5.3^b^	29.6 ± 5.0^b^	77.4 ± 5.0^a^	0.761	< 0.001	0.286
Valine	222.3 ± 45.5^b^	218.9 ± 41.3^b^	622.2 ± 41.8^a^	180.4 ± 45.4^b^	210.8 ± 43.5^b^	675.5 ± 43.8^a^	0.980	< 0.001	0.446
Dispensable	3541.9 ± 361.8^c^	2953.04 ± 319.1^c^	5515.1 ± 319.1^b^	3583.6 ± 338.5^c^	3328.1 ± 319.1^c^	6785.1 ± 319.1^a^	0.043	< 0.001	0.168
Alanine	637.7 ± 117.1^c^	505.6 ± 103.3^c^	1032.7 ± 103.3^b^	776.8 ± 109.5^c^	724.9 ± 103.3^c^	1672.1 ± 103.3^a^	< 0.001	< 0.001	0.050
Asparagine	3.96 ± 0.92^b^	2.99 ± 0.81^b^	6.56 ± 0.81^a^	0.35 ± 0.86^c^	0.20 ± 0.81^c^	2.68 ± 0.81^b^	< 0.001	0.002	0.790
Aspartate	57.5 ± 14.4^b^	41.8 ± 12.96^b^	132.2 ± 13.1^a^	27.4 ± 14.1^b^	22.2 ± 13.4^b^	117.2 ± 13.5^a^	0.099	< 0.001	0.832
Gln + Glu	538.0 ± 84.3^b^	448.8 ± 78.9^b^	832.8 ± 79.9^a^	205.4 ± 89.9^c^	250.1 ± 87.4^c^	450.4 ± 87.9^b^	0.004	< 0.001	0.262
Glycine	1530.5 ± 135.7^cd^	1297.1 ± 119.7^d^	1998.9 ± 119.7^b^	1852.8 ± 126.9^bc^	1611.5 ± 119.7^cd^	2806.2 ± 119.7^a^	< 0.001	< 0.001	0.079
Proline	288.2 ± 50.1^c^	271.1 ± 44.2^c^	675.2 ± 44.2^b^	335.1 ± 46.9^c^	351.7 ± 44.2^c^	921.7 ± 44.2^a^	0.002	< 0.001	0.073
Serine	400.2 ± 51.2^b^	326.5 ± 45.1^b^	658.5 ± 45.1^a^	330.8 ± 47.9^b^	324.6 ± 45.1^b^	696.6 ± 45.1^a^	0.773	< 0.001	0.528
Tyrosine	68.9 ± 14.6^b^	56.4 ± 12.8^b^	154.4 ± 12.8^a^	53.2 ± 13.6^b^	42.1 ± 12.8^b^	115.8 ± 12.8^a^	0.040	< 0.001	0.577
Nonproteinogenic	424.6 ± 65.1^b^	350.3 ± 57.4^b^	670.8 ± 57.4^a^	367.4 ± 60.9^b^	361.7 ± 57.4^b^	653.8 ± 57.4^a^	0.667	< 0.001	0.851
Citrulline	94.4 ± 13.1^bc^	98.0 ± 12.4^bc^	138.1 ± 12.5^a^	70.3 ± 14.3^c^	72.0 ± 14.0^c^	105.1 ± 14.0^ab^	0.096	< 0.001	0.854
Ornithine	104.6 ± 40.4^b^	63.3 ± 35.6^b^	218.4 ± 35.6^a^	53.1 ± 37.8^b^	58.1 ± 35.6^b^	216.9 ± 35.603^a^	0.522	< 0.001	0.765
Taurine	224.6 ± 34.5^c^	185.3 ± 31.8^c^	307.5 ± 32.2^ab^	238.8 ± 35.5^bc^	229.3 ± 34.1^bc^	324.9 ± 34.4^a^	0.493	< 0.001	0.826

Plasma amino acid concentrations represent the mean values at 60 and 90 min during the steady-state phase of the clamp procedure. Values are least-square means ± SE, calculated from 2-factor ANOVA, *n* = 7 (preterm-FAST), 8 (term-FAST), or 9 (preterm-INS, preterm-AA, term-INS, and term-AA) pigs. ^a,b,c,d^ Labeled means in a row without a common superscript letter differ, *P* ≤ 0.05.

Abbreviations: GAB, gestational age at birth; STATE, clamp condition (i.e. FAST, INS, or AA).

### Amino acid transporters

**FIGURE 2:**
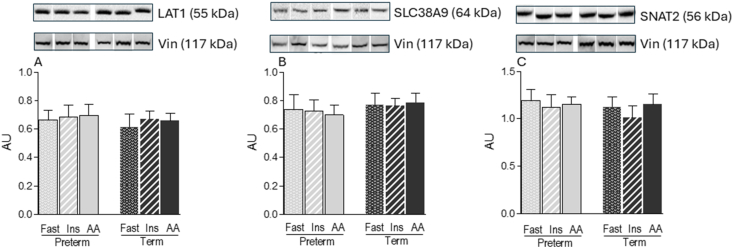
Abundance of amino acid transporters in the longissimus dorsi muscle of preterm and term pigs that were fasted (FAST) or infused with insulin (INS) or amino acids (AA). Abundance of (A) LAT1, (B) SLC38A9, and (C) SNAT2. All samples were normalized to their corresponding vinculin (Vin) values. Representative blots are shown; white lines between bands indicate where images from the same blots were spliced to adjust sample order on the membrane for presentation. Values are means ± SEM; *n* = 5–9. Means with uncommon letters are significantly different, *P* < 0.05.

Both insulin and amino acid signaling pathways converge on mTORC1 to regulate protein synthesis, sharing common downstream effectors. To begin dissecting how prematurity affects the amino acid–sensing branch of the mTORC1 pathway, we assessed the abundance of key amino acid transporters in skeletal muscle. The short-term infusion of insulin or amino acids during the clamp procedures did not alter transporter abundance in either PT or term piglets. Additionally, there were no significant differences in the protein abundance of the L-type amino acid transporter (LAT)1, SLC38A9, or SNAT2 between PT and term groups (Figure 2A–C).

### Intracellular amino acid–sensing components

**FIGURE 3:**
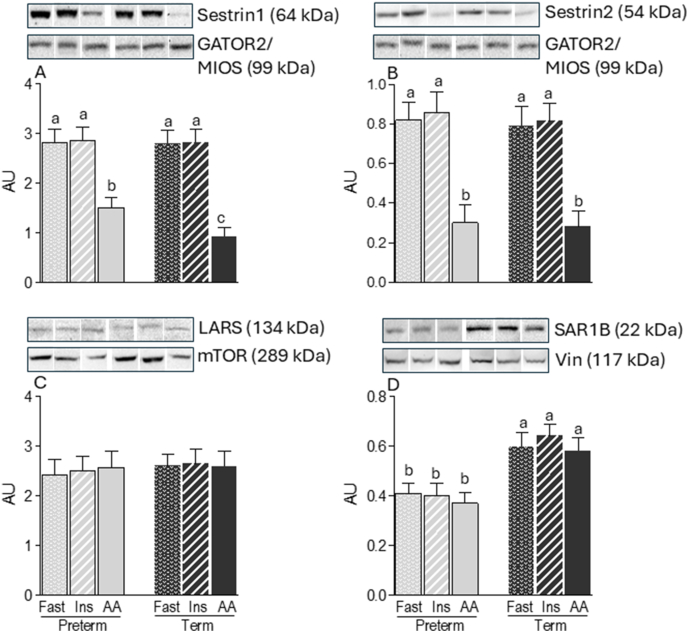
Abundance and protein–protein interactions of leucine sensors in the longissimus dorsi muscle of preterm and term pigs that were fasted (FAST) or infused with insulin (INS) or amino acids (AA). Abundance of (A) Sestrin1-GATOR2, (B) Sestrin2-GATOR2, (C) LARS-mTOR, and (D) SAR1B. Sestrin1/2 and LARS samples were normalized to their corresponding protein partner values, and SAR1B samples were normalized to their vinculin (Vin) values. Representative blots are shown; white lines between bands indicate where images from the same blots were spliced to adjust sample order on the membrane for presentation. Values are means ± SEM; *n* = 5–9. Means with uncommon letters are significantly different, *P* < 0.05.

The abundances of both the Sestrin1-GATOR2 and Sestrin2-GATOR2 inhibitory complexes were reduced by amino acid infusion in both PT (−39% and −75%, respectively) and term (−83% and −77%, respectively) skeletal muscle (*P* < 0.05) (Figure 3A, B), consistent with leucine-induced dissociation of these complexes. However, the abundance of the Sestrin1–GATOR2 complex was higher (+123 %) in amino acid–infused PT piglets than that in term piglets (*P* < 0.05), suggesting reduced leucine sensing in PT skeletal muscle. As expected, insulin infusion had no effect on the abundance of the Sestrin1-GATOR2 and Sestrin2-GATOR2 complexes at either gestational age. The abundance of the LARS-mTORC1 complex was unaffected by amino acid or insulin infusions (Figure 3C). Although our immunoprecipitation followed by immunoblotting analyses did not detect SAR1B-GATOR2 interaction, SAR1B protein abundance was significantly lower in PT piglets than that in term piglets (−52%; *P* < 0.05) (Figure 3D).

**FIGURE 4:**
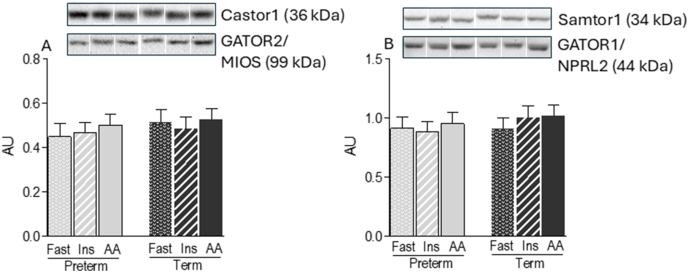
Abundance of protein complexes of arginine and methionine sensors in the longissimus dorsi muscle of preterm and term pigs that were fasted (FAST) or infused with insulin (INS) or AA. Abundance of (A) Castor1-GATOR2 and (B) Samtor-GATOR1. All samples were normalized to their corresponding protein partner values. Representative blots are shown; white lines between bands indicate where images from the same blots were spliced to adjust sample order on the membrane for presentation. Values are means ± SEM; *n* = 5–9. Means with uncommon letters are significantly different, *P* < 0.05.

**FIGURE 5:**
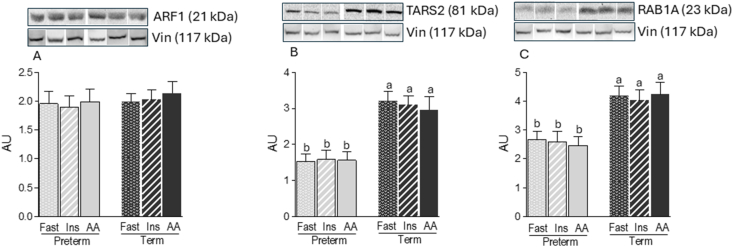
Abundance of glutamine, threonine, and amino acid/branched-chain amino acid sensors in the longissimus dorsi muscle of preterm and term pigs that were fasted (FAST) or infused with insulin (INS) or amino acids (AA). Abundance of (A) ARF1, (B) TARS2, and (C) RAB1A. All samples were normalized to their corresponding vinculin (Vin) values. Representative blots are shown; white lines between bands indicate where images from the same blots were spliced to adjust sample order on the membrane for presentation. Values are means ± SEM; *n* = 5–9. Means with uncommon letters are significantly different, *P* < 0.05.

The abundance of CASTOR1-GATOR2 (arginine sensor) and SAMTOR-GATOR1 (methionine sensor) complexes was not altered by amino acid or insulin infusion (Figure 4A, B). Given the current limited understanding of protein–protein interactions involving glutamine (ARF1), threonine (TARS2), and BCAA (RAB1A) sensors, we evaluated only their abundance in response to prematurity. TARS2 and RAB1A protein concentrations were significantly lower in PT than those in term piglets (-51 and -53 %, respectively; *P* < 0.05) (Figure 5B, C), whereas ARF1 abundance was unaffected (Figure 5A).

### Amino acid signaling

**FIGURE 6:**
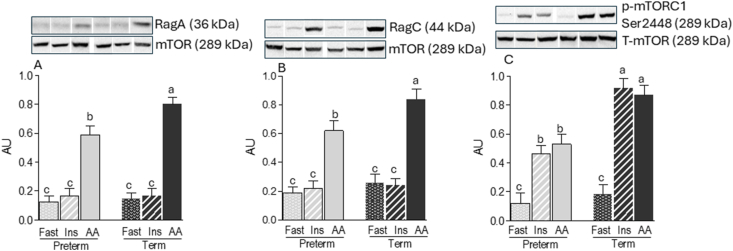
Abundance of Rags protein complexes and mTORC1 phosphorylation in the longissimus dorsi muscle of preterm and term pigs that were fasted (FAST) or infused with insulin (INS) or amino acids (AAs). Abundance of (A) RagA–mTOR (B), RagC–mTOR, and mTORC1 phosphorylation. RagA/C samples were normalized to their corresponding protein partner values. Phospho-mTORC1 samples were normalized to their corresponding total mTOR values. Representative blots are shown; white lines between bands indicate where images from the same blots were spliced to adjust sample order on the membrane for presentation. Values are means ± SEM; *n* = 5–9. Means with uncommon letters are significantly different, *P* < 0.05.

Amino acid infusion increased RagA and RagC complex formation with mTOR in both PT (+120% and +121%, respectively) and term (+235% and +298%, respectively) skeletal muscle (*P* < 0.05) (Figure 6A, B). However, these responses were attenuated in PT piglets compared with that in those born at term (−44% and −48%, respectively; *P* < 0.05), indicating a blunted capacity for amino acid–induced mTORC1 recruitment in PT muscle. As expected, insulin infusion did not alter RagA or RagC complex formation with mTOR in either gestational age group. Both amino acid and insulin infusions increased mTORC1 phosphorylation at Ser2448 in skeletal muscle of both PT (+205 and +212%, respectively) and term (+551 and +515%, respectively) piglets. However, the magnitude of this response was blunted significantly in PT muscle compared with that in term muscle (−44% and −39%, respectively; *P* < 0.05) (Figure 6C). In a previous report from this study, we demonstrated that the downstream targets of mTOR phosphorylation on Ser2448 [10] i.e. phosphorylation of S6K1-Thr389 and 4EBP1-Thr70, were consistent with activation of mTORC1 following Ser2448 phosphorylation.

## Discussion

The nutritional support for PT infants aims to align postnatal growth with that of a normal fetus of the same gestational age [39]. Although the nutritional management and care of PT infants have improved in recent years, most PT infants experience extrauterine growth faltering, especially of lean mass [40]. Understanding the molecular mechanisms by which nutrients regulate muscle growth during the neonatal period is crucial in developing strategies to improve lean accretion. Using neonatal piglets, a relevant translational model for human infants [8,9], we previously demonstrated that the feeding-induced stimulation of protein synthesis in skeletal muscle is attenuated by PT birth [41]. This reduction in the fractional rate of protein synthesis was associated with impaired activation of both insulin- and amino acid–dependent signaling pathways that converge on mTORC1 [41]. However, in that prior study, the postprandial rise in plasma amino acids and insulin was delayed in PT piglets compared with that in term counterparts, despite equivalent relative nutrient intake provided as elemental diets. To distinguish whether the blunted anabolic response was due to delayed systemic nutrient signals or intrinsic defects at the level of skeletal muscle, we used amino acid and insulin-clamp techniques to independently elevate amino acid and insulin concentrations under controlled conditions. Consistent with prior findings, indices of insulin signaling were reduced in skeletal muscle of PT piglets resulting in lower activation of mTORC1 [11]. In the present study, we extended these observations by investigating how prematurity affects the abundance and amino acid–induced activation of key amino acid–sensing components upstream of mTORC1 in the PT muscle.

The molecular mechanisms by which amino acids activate mTORC1 are multifaceted and involve several amino acid–sensing components [16]. Among amino acid transporters, LAT1, SLC38A9, and SNAT2, are well studied in the context of mTORC1 signaling regulation [42]. These transporters are often overexpressed in cancer cells, where elevated amino acid uptake contributes to sustained mTORC1 activation and supports uncontrolled cell growth [43]. This association highlights the functional importance of amino acid transporters in sustaining cellular growth and protein synthesis. Furthermore, the abundance of both LAT1 and SNAT2 was higher in the muscle of 6-d-old than that in 26-d-old pigs, suggesting that the expression of these transporters is developmentally regulated [44]. In the present study, however, prematurity did not alter the protein abundance of LAT1, SLC38A9, or SNAT2 in skeletal muscle. LAT1 and SNAT2 are plasma membrane transporters that facilitate extracellular amino acid uptake, whereas SLC38A9 resides on the lysosomal membrane and mediates luminal arginine sensing to regulate mTORC1 activity [43]. The lack of differences in transporter abundance between PT and term piglets suggests that intracellular amino acid availability is unlikely to be a limiting factor in the impaired mTORC1 signaling observed in PT skeletal muscle.

Nutrient-sensing components, which include amino acid sensors, are a relatively new concept that was proposed to be critical components involved in amino acid–induced activation of mTORC1 [18]. Among them, 3 distinct amino acid sensors for leucine (Sestrin1/2, SAR1B, and LARS) have been identified [19,20,26]. Leucine promotes mTORC1 activation primarily by disrupting the inhibitory association of Sestrins with GATOR2. Evidence from in vivo studies indicates that Sestrin1 is the predominant isoform in skeletal muscle, whereas Sestrin2 abundance is minimal [45]. This difference likely explains why prematurity impaired leucine-induced dissociation of the Sestrin1-GATOR2 complex but not the Sestrin2-GATOR2 complex. The leucine concentration required for dissociation is inversely related to Sestrin abundance: higher Sestrin concentrations demand greater leucine availability to achieve the same degree of mTORC1 activation [18]. Our findings reinforce the central role of Sestrin1 in mediating leucine-dependent mTORC1 activation in skeletal muscle in vivo and suggest that impaired Sestrin1 signaling may contribute to the anabolic resistance observed in PT neonates.

SAR1B has been proposed as a cytosolic leucine sensor that interacts with GATOR2 under leucine-deprived conditions and dissociates upon leucine binding, thereby promoting mTORC1 activation [20]. In mouse skeletal muscle, SAR1B is highly expressed alongside Sestrin1. In vitro studies of myotubes suggest that increasing leucine concentrations may trigger a sequential dissociation of SAR1B and Sestrin1 from GATOR2, eliciting a 2-phase activation of mTORC1. Conversely, leucine starvation induces their sequential binding to GATOR2, resulting in a 2-phase inactivation of mTORC1 [20]. This mechanistic model, however, has not been confirmed in vivo. Despite extensive efforts, we were unable to detect SAR1B-GATOR2 interaction in skeletal muscle. One plausible explanation relates to the reported dissociation constant for leucine-SAR1B interaction (∼2.1 μM) [20], which is substantially lower than the circulating leucine concentrations observed in this study, even under fasting conditions (93.3 μM in PT and 53.9 μM in term piglets). Under such conditions, SAR1B may be constitutively saturated with leucine, potentially obscuring dynamic changes in complex formation. This contrasts with in vitro models, where cells are often completely deprived of amino acids by removing them from the culture medium [18]. Although SAR1B abundance was lower in PT muscle than that in term muscle, the physiological relevance of this reduction remains uncertain given the apparent saturation of SAR1B under physiological leucine concentrations. Collectively, these findings indicate that SAR1B plays a limited role in mediating mTORC1 activation in vivo in neonatal skeletal muscle.

Another leucine sensor, LARS, has been identified as crucial in the leucine-sensing process that activates mTORC1 [26]. However, in our study, we did not detect an effect of amino acid infusion on the abundance of the LARS-mTORC1 complex. This finding is consistent with a human study by Carlin et al. [46], which also showed that essential amino acid ingestion did not affect LARS-mTORC1 association in skeletal muscle. Clearly, the mechanisms by which leucine regulates LARS in vivo need to be further elucidated.

After leucine, arginine is recognized as the second most potent amino acid in stimulating skeletal muscle protein synthesis [47]. Although the effectiveness of arginine in enhancing muscle protein synthesis has been reported in animal and human studies [[48], [49], [50]], the molecular mechanisms by which arginine alters the interaction of its sensor, CASTOR1, with GATOR2 have not been determined in vivo. In this study, we found an interaction between CASTOR1 and GATOR2; however, infusion of an amino acid mixture that raised circulating arginine concentrations ∼2-fold did not affect the abundance of the CASTOR1-GATOR2 complex in the muscle of either PT or term piglets. One possible explanation for the lack of effect of amino acid infusion on an arginine-induced reduction in CASTOR1-GATOR2 complex abundance could be the timing of the analysis. Since our analysis was conducted at only a single point in time, we may have missed a peak of the transient effect of arginine on altering CASTOR1 and GATOR2 interaction. Another consideration is the binding affinity of CASTOR1 for arginine. The reported dissociation constant for the arginine, CASTOR1 interaction is ∼30 μM [18], which is lower than the plasma arginine concentrations observed in our study, even under fasted conditions (192 μM in PT and 51 μM in term piglets). These values suggest that a substantial proportion of CASTOR1 may already be bound to arginine in the fasted state, potentially limiting the extent of further dissociation from GATOR2 upon amino acid infusion. It is important to note, however, although plasma arginine concentrations are above the dissociation constant of the arginine-CASTOR1 interaction, the extent to which circulating arginine concentrations reflect sensor-accessible pools remains uncertain [18]. Further, in vivo studies are needed to clarify the temporal dynamics and tissue-specific regulation of arginine-induced mTORC1 activation, particularly in the context of prematurity.

The anabolic effect of methionine is unique because it is dependent on its metabolite SAM in order to activate mTORC1 [25]. To the best of our knowledge, the metabolic action of SAM in the regulation of the SAMTOR-GATOR1 complex has not been studied in vivo. We found that neither infusion of amino acids that raised circulating methionine concentrations >2-fold nor prematurity altered the abundance of the SAMTOR-GATOR1 complex. Interestingly, an in vivo study showed that methionine supplementation enhanced muscle protein synthesis without affecting the activation of mTORC1 [51]. RAB1A is a sensor for amino acids, especially BCAA [30]. In this study, we found that prematurity suppressed the abundance of RAB1A. Since results from a cancer model study suggested that BCAA are crucial for activation of the RAB1A-mTORC1 axis [29], our finding may indicate that PT birth reduces the sensitivity of skeletal muscle to the BCAA-induced activation of mTORC1. ARF1 and TARS2 may serve as sensors for glutamine and threonine, respectively, as reduced expression of these proteins blunts glutamine- and threonine-induced activation of mTORC1 in vitro [27,28]. Our data showed that TARS2, but not ARF1, abundance in skeletal muscle was reduced by prematurity. To our knowledge, there have been no studies that have examined the function of these amino acid sensors in vivo. Therefore, the physiological relevance of these findings is currently unknown.

Using the clamp technique to independently regulate plasma insulin and amino acid concentrations, we assessed their respective contributions to mTORC1 activation in skeletal muscle. Both insulin and amino acid infusions independently increased mTORC1 phosphorylation at Ser2448, confirming convergence of these pathways on mTORC1. As shown previously [10], downstream of mTORC1, phosphorylation of S6K1-Thr389, 4EBP1-Thr70, and rpS6-Ser235/236/240/244, assembly of the eIF4E–eIF4G complex, and fractional protein synthesis rates in muscle were increased in response to both insulin and amino acid but were blunted by prematurity. However, amino acid, but not insulin, infusion stimulated the formation of RagA-mTOR and RagC-mTOR complexes and suppressed the Sestrin1-GATOR2 and Sestrin2-GATOR2 interactions, providing in vivo evidence that amino acids uniquely activate the amino acid–sensing branch upstream of mTORC1. Previously, we demonstrated that, despite preserved proximal insulin signaling through the insulin receptor and insulin receptor substrate 1, insulin-stimulated phosphorylation of RAC-α serine/threonine-protein kinase (Akt)1 and Akt2 is markedly attenuated in the skeletal muscle of PT piglets [11]. Building on these findings, the present study shows that prematurity also impairs the amino acid–sensing branch of the mTORC1 pathway, compounding the disruption of anabolic signaling and contributing to the diminished capacity for muscle protein synthesis and lean mass accretion observed in PT neonates.

Consistent with the impaired amino acid–mediated activation of mTORC1 signaling in PT muscle, we previously demonstrated that achieving a comparable anabolic response required a leucine dose twice as high in PT piglets as in their term-born counterparts (1600 compared with 800 μmol/kg/h for 1 h every 4 h) [35,52]. Although Boutry et al. [52,53] showed that 800 μmol/kg/h leucine pulses during continuous feeding robustly stimulated mTORC1 signaling and protein synthesis in term piglets, Rudar et al. [35] found that this same dose was insufficient in PT piglets, necessitating a higher leucine concentration to elicit a similar anabolic effect. These findings indicate that although the machinery for leucine-induced protein synthesis is present, PT skeletal muscle exhibits a diminished sensitivity to amino acid stimulation.

Although our understanding of amino acid–mediated mTORC1 activation has advanced considerably in recent years [13,14], most mechanistic insights have been derived from in vitro studies using immortalized or cancer cell lines. The present study, together with our previous work [11], provides in vivo evidence that PT birth impairs both insulin- and amino acid–mediated signaling pathways that converge on mTORC1 in skeletal muscle. Specifically, in this study, we observed reduced leucine responsiveness at the level of Sestrin1-GATOR2 complex dissociation, along with lower abundances of RAB1A (a BCAA sensor and activator of mTORC1) and TARS2 (a threonine sensor and activator of mTORC1) in PT piglets. These impairments were associated with attenuated RagA-mTOR and RagC-mTOR complexes formation and reduced mTORC1 phosphorylation. Collectively, these findings identify specific molecular targets, Sestrin1, TARS2, and RAB1A, which are disrupted by PT birth and likely contribute to the anabolic resistance and impaired lean mass accretion commonly observed in premature infants. These findings also suggest that targeted amino acid supplementation of PT infants could overcome the deficit in lean mass accretion [4] and reduce the lifelong risk of developing obesity and metabolic disease [3].

## Author contributions

The authors’ responsibilities were as follows—AS: designed research, conducted research, analyzed data, and drafted manuscript; ACRdS, KBJ, RDP, MAM: conducted research, analyzed data, reviewed, and edited manuscript; MLF, TAD: designed research, conducted research, analyzed data, and reviewed and edited manuscript; TAD: has primary responsibility for final content; and all authors: have read and approved the final version of the manuscript.

## Data availability

Data described in the manuscript will be made available upon request pending application and approval.

## Funding

This work was supported by the National Institute of Child Health and Development Grants HD-085573 (to TAD) and HD-099080 (to TAD and MLF), USDA Current Research Information System Grant 3092-51000-060 (to MLF), and the Texas A&M AgriLife Institute for Advancing Health Through Agriculture (to TAD).

## Conflict of interest

MLF is a Deputy Editor of *The Journal of Nutrition.* She played no role in the Journal’s evaluation of the manuscript. The other authors report no conflicts of interest.
