# Supplementary material for: Amino Acid Signaling in Skeletal Muscle Is Blunted by Prematurity in a Piglet Model
Source: J Nutr. 2025 Dec 27;156(2):101303. doi: 10.1016/j.tjnut.2025.101303 (PMC12975357; doi:10.1016/j.tjnut.2025.101303)
Supplement: Multimedia component 1 [file mmc1.docx]

**Amino Acid Signaling in Skeletal Muscle Is Blunted by Prematurity in a Piglet Model**

Antonio C. Ramos dos Santos,^1,†^ Agus Suryawan,^2,†^ Ki Beom Jang,^3^ Rosemarie D. Parada,^1^ Mahmoud A. Mohammad,^2^ Marta L. Fiorotto,^2^ Teresa A. Davis^1,2,4,^*

Supplementary Table 1. Plasma concentrations of glucose (mg·dL^-1^), insulin (µU·mL^-1^), and branched-chain amino acids (BCAA; µmol·L^-1^) in preterm and term pigs during euinsulinemic-euaminoacidemic-euglycemic (FAST), hyperinsulinemic-euaminoacidemic-euglycemic (INS), or euinsulinemic-hyperaminoacidemic-euglycemic (AA) clamp conditions

|  | Preterm | | |  | Term | | |  | *P*-value | | |
| --- | --- | --- | --- | --- | --- | --- | --- | --- | --- | --- | --- |
|  | FAST | INS | AA |  | FAST | INS | AA |  | GAB | STATE | GAB×STATE |
| Glucose, mg·dL^-1^ | 52.7 ± 3.9 | 47.8 ± 3.4 | 53.4 ± 3.4 |  | 53.7 ± 3.6 | 61.1 ± 3.4 | 61.7 ± 3.4 |  | 0.012 | 0.452 | 0.240 |
| Insulin, µU·mL^-1^ | 1.0 ± 8.3^b^ | 105.1 ± 7.4^a^ | 2.9 ± 7.4^b^ |  | 1.4 ± 7.8^b^ | 101.3 ± 7.4^a^ | 2.1 ± 7.4^b^ |  | 0.826 | < 0.001 | 0.960 |
| BCAA, µmol·L^-1^ | 319.7 ± 24.5^b^ | 319.4 ± 21.6^b^ | 1055.6 ± 21.6^a^ |  | 297.6 ± 22.9^b^ | 311.9 ± 21.6^b^ | 1063.4 ± 21.6^a^ |  | 0.692 | < 0.001 | 0.803 |

Plasma concentrations of glucose, insulin, and BCAA represent the mean values at 60, 90, and 120 minutes during the steady-state phase of the clamp procedure. Values are least-square means ± SE calculated from two-factor ANOVA, n = 7 (Preterm-FAST), 8 (Term-FAST), or 9 (Preterm-INS, Preterm-AA, Term-INS, and Term-AA) pigs. Labeled means in a row without a common superscript letter differ, *P* ≤ 0.05. AA, euinsulinemic-hyperaminoacidemic-euglycemic clamp; BCAA, branched-chain amino acids; FAST, euinsulinemic-euaminoacidemic-euglycemic clamp; GAB, gestational age at birth; INS, hyperinsulinemic-euaminoacidemic-euglycemic clamp; STATE, clamp condition (i.e., FAST, INS, or AA).
